# Mass transfer analysis and kinetic modeling of ultrasound-assisted osmotic dehydration of kiwifruit slices

**DOI:** 10.1038/s41598-023-39146-x

**Published:** 2023-07-22

**Authors:** Fakhreddin Salehi, Rana Cheraghi, Majid Rasouli

**Affiliations:** 1 Department of Food Science and Technology, Bu-Ali Sina University, Hamedan, Iran; 2 Faculty of Agriculture, Bu-Ali Sina University, Hamedan, Iran

**Keywords:** Biophysics, Plant sciences

## Abstract

Ultrasound treatments (sonication) in combination with osmotic dehydration process accelerate the rate of moisture removal from the fruits or vegetables pieces and decrease the dehydration duration. The purpose of this study was to examine the influence of ultrasound-assisted osmotic dehydration (UAOD) on mass transfer kinetic (soluble solids gain and moisture loss) of kiwifruit slices. The UAOD process was performed using 20, 30, and 40% sucrose solutions in ultrasonic bath (40 kHz, 75 and 150 W) for 10, 20, 30, 40, 50, 60, 70, and 80 min. After treatments, processed kiwifruit slices were dried at 70 °C using hot air oven. UAOD process reveals that mass reduction, soluble solid gain, moisture loss and rehydration capacity affected by treatments time, sucrose solution concentration and sonication power. The results showed that the UAOD treatment increased moisture loss and soluble solids gain. Furthermore, kiwifruit slices treated with higher ultrasound intensity (150 W) showed reduced dehydration duration (higher water loss), improved dehydration rate, and increased effective moisture diffusivity (D_eff_). The D_eff_ determined by Fick's second law was varied from 9.05 × 10^–11^ to 29.28 × 10^–11^ m^2^s^-1^. The experimental data of dehydration curve of kiwifruit slices were fitted to different thin-layer equations and the Page equation with empirical constants was the best describing the of kiwifruit slices dehydration.

## Introduction

Ultrasound (sonication) and its combined treatment reduced color changes, preserved firmness, removed entrapped air bubbles, increased and inhibited enzymatic activity, and also saved nutritional components such as total phenols, total flavonoids, anthocyanins, and ascorbic acid^1–4^. Additionally, Jalilzadeh, et al.^5^ found that sonication had a significant effect on the sensory attributes of cheese samples. Also, Chen and Fan^6^ results confirmed that the combination of sonication and modified atmosphere was an effective preservation method to improve the preservation quality of freshly cut lettuce. Osmotic dehydration is often used to partially remove moisture from fruits and vegetables tissues and enrich them with functional compounds. The high mass transfer resistance of plant cell membranes presents an important barrier to moisture diffusion during osmotic dehydration^7,8^. The combination of the sonication process with the osmotic dehydration technique commonly named ultrasound-assisted osmotic dehydration (UAOD) can more improve the drying process efficiency by increasing the water transfer rates, decreasing the drying time and energy utilization, and improving dried products quality^9–11^. Li, et al.^12^ studied the impact of UAOD pretreatment on the dehydration rate of Sanhua plum. The authors reported that the use of ultrasonic pretreatment enhanced mass transfer during osmotic dehydration of Sanhua plum. The solid gains of the control, 0.45 W/g (30% power), 0.90 W/g (60% power), and 1.35 W/g (90% power) groups were 7.44%, 9.06%, 10.81%, and 12.64%, respectively, which indicated that ultrasound pre-treatment contributed to added solid gain of Sanhua plum. In addition, the original water loss of the control, 0.45 W/g, 0.90 W/g, and 1.35 W/g groups were 4.01%, 4.29%,4.89%, and 6.16%, respectively. In another study, the impact of UAOD pretreatments on dehydration of pulsed fluidized bed microwave freeze-dried strawberries was studied by Jiang, et al.^13^. Their research demonstrated that the application of UAOD treatments can lead to a 10% reduction in drying time and an increase in the rate of dehydration. The results by Azarpazhooh, et al.^14^ showed that ultrasonic treatment (20 kHz, 50 °C, and 60 min) had a considerable impact on the physicochemical properties (phenolic compounds, anthocyanin compounds, color indexes, water loss, adsorption of solids, dehydration index, and weight loss) of *Aloe vera* gel. The UAOD treatment enhanced the amount of water loss (52.94%) and solids gain (5.65%), compared with other techniques. In addition, the dried Aloe vera gel pre-treated, had higher anthocyanin compounds and its color was lighter and reddened. The results of the sensory evaluation of the samples showed that this pretreatment improved the color, texture and appearance of the product.

Advanced processes must be combined with energy efficient processes to yield high quality products^15^. In our previous study^16^, we used ultrasound technique for improving osmotic dehydration process of apple slices. The goal of this study was to explore the impacts of various ultrasonic intensities (0, 75 W, and 150 W) and osmotic solution concentrations (20, 30, and 40%) on the mass transfer kinetic (water loss and solid gain) of kiwifruit slices. In addition, the impacts of UAOD process on the mass reduction, rehydration capacity and D_eff_ during the treatment conditions were examined.

## Materials and methods

### Preparation of kiwifruit slices

The collection of kiwifruit in this research was done in accordance with the law and formal approval of the Iranian National Standards Organization. Samples of kiwifruits (*Actinidia deliciosa*) were purchased from the fruit garden market at Sari, Mazandaran, Iran. The fresh and uniform-size kiwifruits with no external damage were selected, and with the aid of an industrial slicer (food slicer, model AF-23, Girmi, Italy) and a cylindrical shape mold cut into 5 mm-thick samples. The initial kiwifruit slices moisture content (MC) was 84% w.b. (moisture determination was performed in a Shimaz oven, Iran, at 105 °C for 4 h).

### Ultrasound-assisted osmotic dehydration procedure

**Figure 1:**
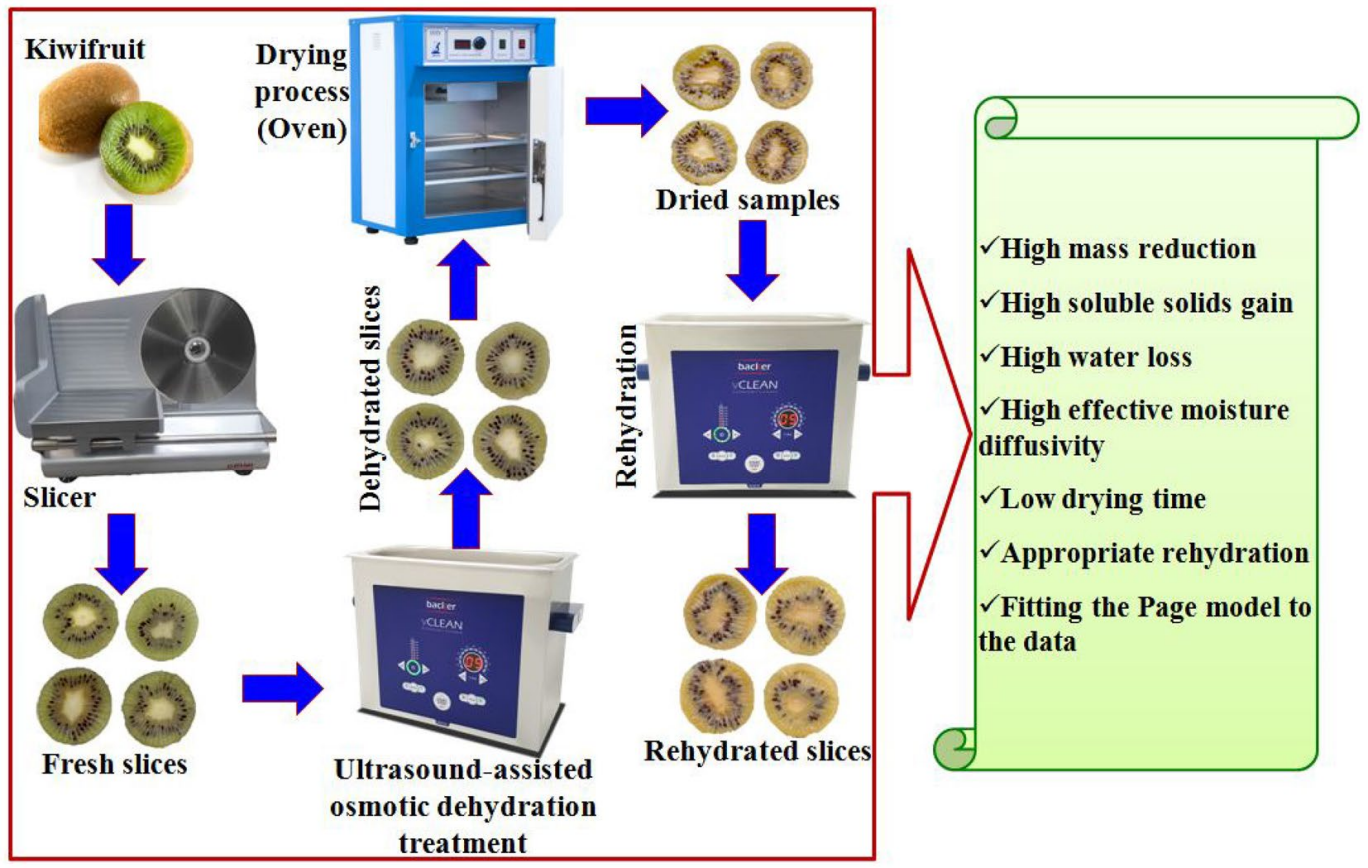
Schematic of the ultrasound-assisted osmotic dehydration procedure of kiwifruit slices.

To apply the sonication treatments on the kiwifruit slices, a Backer vCLEAN1-L6 ultrasonic bath (Iran) was employed with a frequency of 40 kHz and a power of 75 and 150 watts (Fig. 1). The tank of the device was filled with 4 L of 20, 30, and 40% (w/w) sucrose solutions and, then, after the temperature of the solutions reached to 50 °C, the kiwifruit slices were placed directly in the bath. In this investigation, we tried to keep the temperature constant in all processes. The equipment used for this study is equipped with precision thermometers and thermocouples, and temperature variations during the process were minimal. Following this, to determine the mass of the slices a digital scale (Kia Laboratory Weighing, model SL1000, with an accuracy of ± 0.01 g, Iran) was used. Subsequently, after the termination of each experiment, kiwifruit slices were removed from the bath with forceps and wrap it immediately with clean tissue paper to remove excess moisture. Finally, the treated samples were placed in an oven in single-layer (Shimaz, Iran) at 70 °C (until reaching a constant mass). At each step, sonication treatments were applied to the kiwifruit slices in three replicates and 8 time intervals (10, 20, 30, 40, 50, 60, 70, and 80 min).

### Mass transfer

The dehydrated kiwifruit slices were analyzed with respect to MRe (mass reduction), SG (soluble solids gain) and WL (water loss) using Eqs. (1), (2) and (3)^16^, respectively:

1 $${\text{MRe}} = \frac{{A_{0} - A_{t} }}{{A_{0} }} \times 100$$

2 $$SG = \frac{{S_{t} - S_{0} }}{{A_{0} }} \times 100$$

3 $$WL = \frac{{W_{0} - W_{t} }}{{A_{0} }} \times 100 = \frac{{W_{0} - (A_{t} - S_{t} )}}{{A_{0} }}$$

where A_0_ is the initial kiwifruit slice mass (g) before treatment; A_t_ is the final kiwifruit slice mass (g) after treatment; W_0_ is the fresh kiwifruit slice water content in wet basis before treatments (g); W_t_ is the final samples water content in wet basis after treatment (g); S_0_ is the initial solid contents (g) before treatment; and S_t_ is the final kiwifruit slice solid contents (g) after treatment.

### Rehydration ratio (RR) of kiwifruit samples

The process of reintroducing moisture to dried fruit or vegetables product to reach similar moisture levels as in its initial situation is called rehydration^17^. Dried kiwifruit slices were weighed (M_0_) and immersed for 20 min in water (200 mL) at 50 °C. Then, the moisture was drained during two minutes and the samples were weighed again (M_r_). The rehydration ratio was determined by the following formula (Eq. (4)), as the ratio of the final mass of samples (rehydrated kiwifruit slices) over the initial dried kiwifruit slices mass (after oven)^18^. The measurements were repeated three time for each sample.

4 $$RR = \frac{{M_{r} }}{{M_{0} }} \times 100$$

### Kinetics modeling

The Page, Newton, Midilli, Logarithmic, Verma, and Two terms mathematical models were selected to describe kiwifruit slices dehydration behavior (Table 1)^19^. Matlab software (version R2012a) was employed to calculation the constants of these models. The following equation was employed to determine the moisture ratio (MR) parameter:

5 $$MR = \frac{{M_{t} }}{{M_{0} }}$$

where MR is the water removal factor described by the ratio of the water content of kiwifruit slices at time t (M_t_) over the initial water content (M_0_). In addition, SSE (sum of squares due to error), RMSE (root mean squared error), and R^2^ values were calculated to establish the degree of correlations between the empirical and the estimated data. The best fitting between the empirical data and the correlation is obtained when a amalgamation of the highest R^2^ value and the lowest SSE or lowest RMSE.

**Table 1:** Mathematical models applied to the moisture ratio (MR) values.

Model No	Model	Equation
1	Page	_MR= *exp*(-ktn)_
2	Newton	_MR= *exp*(-kt)_
3	Midilli	_MR= a*exp*(-ktn)+bt_
4	Logarithmic	_MR= a*exp*(-kt)+c_
5	Verma	_MR= a*exp*(-kt)+(1-a)*exp*(-gt)_
6	Two term	_MR= a*exp*(-k0tn)+b*exp*(-k1t)_

### Effective moisture diffusivity (D_eff_)

The D_eff_ is an important transport property in fruit and vegetable and other materials dehydration processes modeling^20,21^. The rate of water removal can be given by the Fick's second law. We used the Eq. (6) for determining the D_eff_ values of kiwifruit slices during osmotic dehydration:

6 $$D_{eff} = - \frac{{Slope(K) \times 4 \times L^{2} }}{{\pi^{2} }}$$

where, D_eff_ is the effective moisture diffusivity (m^2^/s), L is the half-thickness of the kiwifruit slice (m), and slope(k) is the slope of lnMR data versus dehydration time of kiwifruit slices (s)^16,22^.

## Results and discussion

### Mass reduction

**Figure 2:**
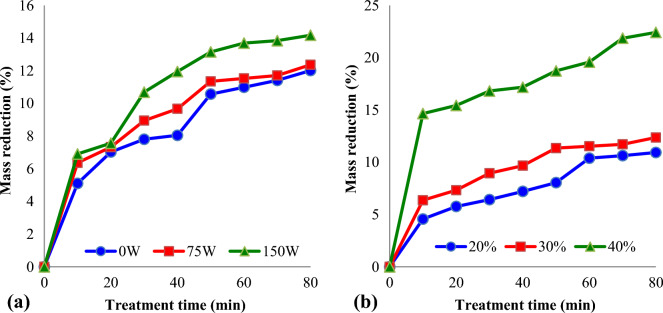
Variations of mass reduction (%) of kiwifruit slices during osmotic dehydration at different: (**a**) ultrasound intensity (concentration = 30%); (**b**) sucrose solution concentration (ultrasound intensity = 75W).

The reduction in mass with decreasing water content is important in dewatering as it directly relates to reducing procedure duration or cost to the final product^8,23^. The changes in mass reduction (%) of kiwifruit slices during UAOD treatments were seen in Fig. 2a. As shown in the figure, ultrasound intensity plays an important role in mass reduction. It was considered that mass reduction increased with the increasing in ultrasound intensity. When sonication power increased to 75 W and 150 W, reductions in kiwifruit slices mass (after 80 min) were 12.37% and 14.17%, respectively (concentration = 30%). Figure 2b shows the influence of sucrose levels on the mass reduction (%) of kiwifruit slices during osmotic dehydration. It was observed that mass reduction increased with the increasing in sucrose levels from 20 to 40%. Bozkir and Ergün^24^ examined the effects of applying ultrasonic pretreatment (35 kHz/10, 20, 30 min) and osmotic dehydration on the hot air drying behavior and quality of persimmons. Results showed that water loss, solid gain, and weight reduction increased with the duration of ultrasonic treatment. In addition, the drying rate (33%) and effective diffusivities (46%) increased with increasing sonication time.

### Soluble solids gain

**Figure 3:**
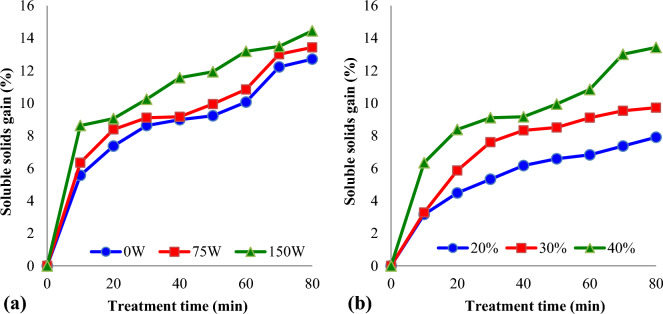
Variations of soluble solids gain (%) of kiwifruit slices during osmotic dehydration at different: (**a**) ultrasound intensity (concentration = 40%); (**b**) sucrose solution concentration (ultrasound intensity = 75W).

Influence of ultrasound intensity and treatment time on the soluble solids gain of UAOD treated kiwifruit slices is reported in Fig. 3a. As shown in the figure, soluble solids gain percentage of UAOD treated kiwifruit slices (75W and 150W) was found to be higher than osmotic dehydrated kiwifruit slices (0W). This may be due to the changes inside the kiwifruit slices structure caused by cavitation and microstreams (effects of ultrasound)^25^. In the UAOD treated (ultrasound intensity = 150W) the kiwifruit slices gained 9.11% of sucrose after 80 min and in the osmotic dehydration without sonication treatment, the kiwifruit slices gained 3.28% of sucrose in the 80 min (concentration = 20%).

The driving force for the diffusion of moisture from the fruit and vegetable tissue into the osmotic solution is provided by the higher osmotic pressure of the hypertonic solution (sucrose). Diffusion of moisture involves simultaneous counter diffusion of solutes from osmotic solution into the tissue^26,27^. Also, the greater the osmotic pressure difference, the greater the flux of water (cell to solution), which is usually accompanied by an increased backflow of dissolved solids (solution to cell)^25^. Figure 3b show the influence of sucrose levels on the soluble solids gain (%) of dehydrated kiwifruit slices. The soluble solids gain of kiwifruit slices increased from 6.76% to 13.53% with increasing sucrose levels from 20 to 40%. Additionally, with increasing treatments duration, the soluble solids gain of kiwifruit slices increased. It could be concluded that the samples submitted to osmotic dehydration treatment (with or without sonication) for ten minutes and for eighty minutes have the lowest and the highest solids gain, respectively. Shamaei, et al.^28^ investigated the effect of osmotic methods with or without ultrasound on soluble solids gain and moisture loss of cranberries. They observed that using ultrasound for osmotic dehydration of cranberries resulted in faster soluble solids gain and moisture loss. The results of El-Aouar, et al.^26^ study showed that, considering the same osmotic pressure for both osmotic agents (sucrose and corn syrup), the values obtained for mass reduction, water loss, and solids gain for dehydration of papaya (*Carica papaya* L.) in sucrose solutions were higher than those obtained in corn syrup solutions, due to their high viscosity and polysaccharide content.

### Water loss

**Figure 4:**
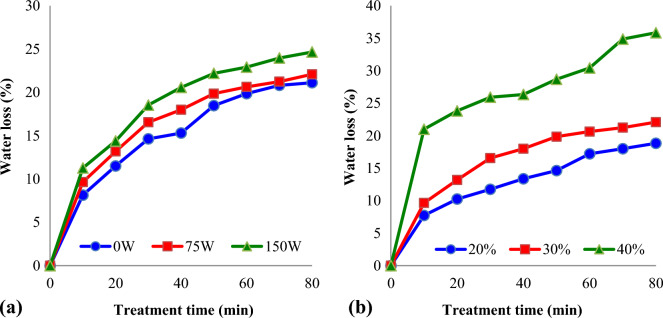
Variations of water loss (%) of kiwifruit slices during osmotic dehydration at different: (**a**) ultrasound intensity (concentration = 30%); (**b**) sucrose solution concentration (ultrasound intensity = 75W).

Several studies are conducted using osmotic dehydration and further improvement of drying performance was done by integrating ultrasound technique for drying of fruit and vegetable^27^. The changes in water loss (%) of kiwifruit slices during UAOD treatments as function of ultrasound intensity were seen in Fig. 4a. As shown in the figure, sonication ultrasound intensity plays a major function in moisture loss. It was verified that moisture loss increased with the increasing in ultrasound intensity. When sonication power increased to 75 W and 150 W, reductions in kiwifruit slices moisture content (after 80 min) were 35.85% and 41.83%, respectively (concentration = 40%). Similar findings were reported by Fernandes, et al.^29^, where after 90 min of sonication treatment with the frequency of 25 kHz, papaya (*Carica papaya* L.) lost 11.92 ± 1.4% of initial water content. Also, this study results demonstrate that the D_eff_ increases after treatment of sonication causing a decrease of about 16% in the drying time of samples. In Prithani and Dash^30^ study, the impact of ultrasonic application (25 kHz for 20 min) on osmotic dehydration (300 min in 60%, sucrose) of kiwi slices were examined. The osmotic dehydration procedure demonstrated a quick initial water reduction followed by a progressive reduction in the velocity in the long periods. Barman and Badwaik^31^ studied on the effect of ultrasound and centrifugal force on carambola (*Averrhoa carambola* L.) slices during osmotic dehydration. The results showed that with increasing in treatment time the water loss, rehydration ratio were increased. The controlled samples showed 68.14% water loss in carambola slices, while, the sample having 30 min ultrasonic treatment showed 73.76% water loss.

Water loss during the osmotic dehydration process is primarily affected by the type of osmotic solution and its concentration. Figure 4b shows the influence of sucrose concentration on the moisture loss of kiwifruit slices during osmotic dehydration. It could be concluded that the moisture loss of slices was increased with increasing in the sucrose levels from 20 to 40%. The time required to reduce the moisture content of kiwifruit slices about by 18% (w.b.) was found as 80, 40 and 10 min for 20%, 30% and 40%, respectively (ultrasound intensity = 75W).

### Rehydration ratio

**Figure 5:**
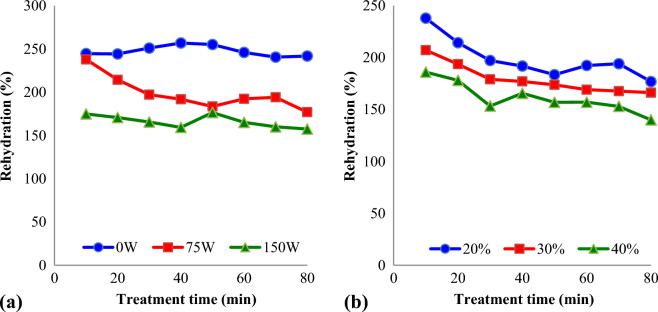
Variations of rehydration (%) of dried kiwifruit slices treated by different:** a**) ultrasound intensity (concentration = 20%); (**b**) sucrose solution concentration (ultrasound intensity = 75W).

The changes in rehydration ratio of kiwifruit slices during UAOD treatments as function of ultrasound intensity were seen in Fig. 5a. As shown in the figure, the ultrasound intensity has a considerable effect on the rehydration ratio of the UAOD treatments kiwifruit slices. These results can be explaining with the contraction of the kiwifruit slices, which is cause by sonication treatment. Also, Fig. 5b shows the impact of sucrose concentrations on the rehydration ratio of dried kiwifruit slices. The factor which influences on the rehydration ratio of kiwifruit slices is the structure of the dried samples and conditions of UAOD treatment includes ultrasound intensity, sucrose concentration, and treatment time. The rehydration ratio of kiwifruit slices reduced from 204.1% to 158.8%, by increasing sucrose concentration from 20 to 40%. Similarly, Farooq, et al.^32^ reported that the rehydration ratio of Rambutan decreased with increasing osmotic solution concentration. Shekar and Javadi^33^ observed a decrease in rehydration rate when using UAOD compared to control samples, and observed a greater reduction in UAOD samples than in osmotically dehydrated samples.

### Dehydration kinetics

Among examined equations, the Page model (Table 1) is given better prediction than the other models and satisfactorily described dehydration kinetics of kiwifruit slices. This model considers the effects of dehydration conditions provided good fitting of the predicted moisture ratio with the highest R^2^, and the lowest SSE and RMSE values. The constants (k and n) of the kiwifruit slices dehydration kinetics model (Page model) at various dehydration conditions are presented in Table 2. The statistical parameters used for selecting the samples drying kinetics model (R^2^, SSE and RMSE) are also presented in the table. The R^2^ values for all dehydration conditions were between 0.9753 and 0.9962. In addition, the SSE and RMSE values for all dehydration conditions were between 0.0001 and 0.0032, and 0.0047 and 0.0216, respectively.

**Table 2:** Parameters of Page model describing the kinetics of osmotic dehydration rate of kiwifruits slices.

Ultrasound intensity (W)	Concentration (%)	k	n	R^2^	SSE*	RMSE**
0	20	0.0130	0.6127	0.988	0.0003	0.0065
75	20	0.0294	0.4866	0.996	0.0001	0.0047
150	20	0.0484	0.3778	0.991	0.0004	0.0072
0	30	0.0335	0.5018	0.992	0.0004	0.0079
75	30	0.0495	0.4225	0.989	0.0007	0.0097
150	30	0.0539	0.4322	0.994	0.0004	0.0080
0	40	0.1184	0.3241	0.982	0.0021	0.0171
75	40	0.1189	0.3381	0.975	0.0032	0.0216
150	40	0.1390	0.3604	0.991	0.0017	0.0158

*The sum of squares due to error (SSE).

**Root mean squared error (RMSE).

### Comparisons of experimental and predicted

**Figure 6:**
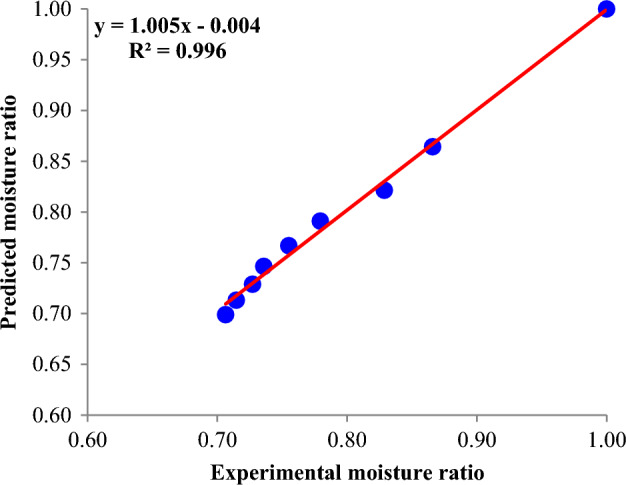
Evaluation of fitted the data by the Page model with empirical results (ultrasound intensity = 150 W and sucrose concentration = 30%).

The dehydration kinetics model's validation through the moisture ratio plot on the experimental results is presented in Fig. 6. The Page model was found to match the experimental moisture ratio data (at various dehydration conditions) very closely with the maximum SSE and RMSE less than 0.0032 and 0.0216, respectively. The coefficient of determination for predicting moisture ratio of kiwifruit slices for the ultrasound intensity equal 150 W and sucrose concentration equal 30% was 0.996. The model prediction for moisture ratio of kiwifruit slices from this study is as good as the results of the other researcher. These results were consistent with the results from dehydration kinetic fitting of apple slices^16^. The effect of UAOD with pulsed fluidized bed microwave freeze drying of Chinese yam was examined Li, et al.^34^. The UAOD pre-treatment decreased the drying duration and saved energy. Also, in this study, the Page model was successfully fitted to the dehydration curves.

### Effective moisture diffusivity (D_eff_)

D_eff_ values were calculated for the different dehydration conditions. The influence of ultrasound intensity and sucrose concentration on the D_eff_ values of kiwifruit slices during osmotic dehydration are reported in Table 3. In this study, the D_eff_ determined by Fick's second law was varied from 9.05 × 10^–11^ to 29.28 × 10^–11^ m^2^s^-1^. The average D_eff_ values improved from 1.07 × 10^–10^ to 2.43 × 10^–10^ m^2^s^-1^ with enhancing ultrasound intensity from 0 to 150 W. Additionally, the average D_eff_ values increased with addition sucrose concentrations and they were equal to 1.07 × 10^–10^, 1.46 × 10^–10^ and 2.43 × 10^–10^ m^2^s^-1^ for 20, 30 and 40%, respectively. A greater difference in osmotic pressure causes a higher diffusion of water (higher D_eff_ value) from the fruit cell to the osmotic solution. Similar results were reported by some researchers. Fernandes and Rodrigues^35^ confirmed that the D_eff_ of some fruits increased after sonication treatment. Salehi, et al.^16^ used a kinetic model to predict the water loss data at each time during the UAOD procedure for apple slices. The D_eff_ during this procedure ranged from 1.48 × 10^–10^ m^2^s^-1^ to 4.62 × 10^–10^ m^2^s^-1^.

**Table 3:** The impacts of sonication power levels and osmotic solution concentrations on the effective moisture diffusivity (D_eff_) values of kiwifruit slices during osmotic dehydration.

Ultrasound intensity (W)	Concentration (%)	Slope	D_eff_ (m^2^/s)	r
0	20	0.0022	9.05E−11	0.965
75	20	0.0028	1.17E−10	0.957
150	20	0.0028	1.15E−10	0.934
0	30	0.0033	1.39E−10	0.951
75	30	0.0033	1.39E−10	0.920
150	30	0.0038	1.59E−10	0.920
0	40	0.0050	2.10E−10	0.902
75	40	0.0054	2.26E−10	0.903
150	40	0.0070	2.93E−10	0.914

## Conclusion

The results showed that the ultrasound intensity and sucrose solutions concentrations influenced on the mass reduction, soluble solids gain, moisture loss and rehydration kinetics of kiwifruit slices. Increasing the sonication power, increasing the treatment time, and increasing the concentration of the sucrose solution increased the percentage of mass loss and thus the amount of water removed from the kiwifruit slices. It was found that the application of ultrasound during osmotic dehydration resulted in higher water loss and soluble solids gain. The Page model was found to match the experimental data very closely with the maximum SSE and RMSE less than 0.0032 and 0.0216, respectively. The D_eff_ values of kiwifruit slice samples during osmotic dehydration were determined in the range of 9.05 × 10^–11^ and 29.28 × 10^–11^ m^2^s^-1^; and the D_eff_ values were increased from 1.47 × 10^–10^ to 1.89 × 10^–10^ m^2^s^-1^ with increasing ultrasound intensity levels from 0 to 150 W and they were increased from 1.07 × 10^–10^ to 2.43 × 10^–10^ m^2^s^-1^ with increasing sucrose levels from 20 to 40%. Overall, the optimum operating condition for UAOD process of kiwifruit slices was found to be sucrose concentration of 40%, and ultrasound intensity of 150W (40 kHz). This study did not discuss changes in sample temperature during processing. Therefore, future studies are recommended to investigate the effect of changing sample temperature during UAOD on the mass transfer rate and final product quality.

## Data Availability

All data generated or analysed during this study are included in this published article.
